# Dressings Combined with Injection of Meglumine Antimoniate in the Treatment of Cutaneous Leishmaniasis: A Randomized Controlled Clinical Trial

**DOI:** 10.1371/journal.pone.0066123

**Published:** 2013-06-24

**Authors:** Alireza Khatami, Rezvan Talaee, Makan Rahshenas, Ali Khamesipour, Pedram Mehryan, Sepideh Tehrani, Yahya Dowlati, Alireza Firooz

**Affiliations:** 1 Center for Research and Training in Skin Diseases and Leprosy, Tehran University of Medical Sciences, Tehran, Iran; 2 Department of Dermatology, Kashan, University of Medical Sciences, Kashan, Iran; 3 Faculty of Medicine, Islamic Azad University, Tehran Medical Branch, Tehran, Iran; 4 Department of Dermatology, Faculty of Medicine, Islamic Azad University, Tehran Medical Branch, Tehran, Iran; The Ohio State University, United States of America

## Abstract

**Background:**

Cutaneous leishmaniasis (CL) is a neglected infectious disease and a major health problem in several developing countries. Despite some reasonable explanation for their potential benefits, there is only trace evidence regarding the role of dressings in the treatment of CL.

**Methods:**

This randomized, assessor-blind, controlled, clinical trial was conducted in an endemic area for CL caused by *Leishmania major* in Iran to assess the efficacy of administration of weekly intralesional meglumine antimoniate (i.l.MA) either alone or combined with application of a silver or a non-silver polyester dressing on their lesions for 6 weeks. After screening of 241 patients with CL lesions, 83 eligible patients with 158 lesions were randomly allocated in three arms of the study. Eligibility criteria included parasitologically confirmed CL, age of 12 to 60 years; willingness to participate, duration of lesion<3 months, number of lesions<5, largest ulcer diameter<5 cm. Pregnant or lactating women were excluded. The primary outcome was absolute risk reduction (ARR) based on the proportion of complete healing, which was defined as more than 75% reduction in the size of the lesion compared with baseline in each group at the termination of treatment and 1 month later.

**Findings:**

ARR (95% Confidence Interval [CI]) in i.l.MA versus i.l.MA+non-silver dressing groups was 5.98% (−7.07% to 20.25%), between i.l.MA versus i.l.MA+silver dressing groups was −0.23% (−13.53% to 14.82%), and between i.l.MA+non-silver dressing versus i.l.MA+silver dressing groups was −6.21%(−18.28% to 6.52%) after 6 weeks of treatment. ARR (95% CI) in i.l.MA versus i.l.MA+non-silver dressing groups was −2.22% (−22.12% to 18.10%), between i.l.MA versus i.l.MA+silver dressing groups was 3.64% (−15.36% to 22.82%), and between i.l.MA+non-silver dressing versus i.l.MA+silver dressing groups was 5.86% (−12.86% to 24.31%) 1 month later.

**Conclusion:**

It could not be demonstrated that the efficacy of i.l.MA was improved by either dressing.

**Trial Registration:**

Iranian Registry of Clinical Trials (IRCT.ir) IRCT138707201166N2.

## Introduction

Leishmaniasis is caused by different species of intracellular protozoan, *Leishmania* and transmitted by the bite of infected sand flies. Leishmaniasis is endemic in 88 countries, mostly developing ones. Worldwide, 350 million individuals are at risk of leishmaniasis and 1.5–2 million new cases of leishmaniasis occur each year, of which 75% are cutaneous [1]. Ninety percent of all cutaneous leishmaniasis (CL) cases occur in 7 countries: Afghanistan, Algeria, Brazil, Iran, Peru, Saudi Arabia and Syria. It is considered as a major health problem in 14 countries [2]. Iran is endemic for CL and almost all CL cases in Iran are caused by either *L. tropica* or *L. major* [3]. CL has been reported from all provinces of Iran and is endemic in many of them. In general, CL is a neglected infectious disease, which is a major health problem in several developing countries including endemic areas in Iran [1], [4]–[7].

Many treatment modalities have been used in the treatment of CL, but pentavalent antimonies are considered as the first line drugs for treatment so far. They have to be administered only as intramuscular, intravenous or intralesional injections and could be associated with severe side effects and significant discomfort [8]. There is no vaccine available for prevention of CL for general human use [9]. Two published systematic reviews demonstrated that there is a lack of strong evidence regarding effective, safe and inexpensive treatments for CL [8], [10].

It is logical to imagine that bringing all aspects of wound management paradigm into consideration for treating ulcerative lesions of CL may improve the results of any intervention that are used in the treatment of CL cases. Wound management paradigm includes treating the cause, wound bed preparation and patient related concerns. Wound bed preparation contains debridement, control of inflammation or infection and moisture balance [11].

Unfortunately, the conventional treatments of CL tend to concentrate only on the treatment of the causative protozoa and little attention has been paid to other aspects of wound management in this disease. In the majority of randomized controlled clinical trials (RCTs) in which the efficacy of different therapeutic interventions for acute Old World cutaneous leishmaniasis have assessed, principles of wound management has been ignored [12], [13]. Sadeghian et al. have reported a decrease in the therapeutic efficacy of i.l.MA injections in CL lesions with secondary bacterial infection [14]. It has been recommended to use moisturizing dressings (polyurethane containing types) in the treatment of the lesions of CL [15]. Almost all chronic wounds are colonized with different microorganisms and it is well known that when the bacterial burden of the wound gradually increases, it could interfere with normal wound healing, deteriorate the inflammation and pain of the wound, and results in more complex management of it [12]–[14].

Use of appropriate antimicrobial dressing for ulcerative CL may improve wound healing by reducing the bacterial burden of the wound and providing clean moist environment.

Silver dressing can improve local wound care through provision of infection control and moisture balance [16]. Silver exerts its antimicrobial effects through several mechanisms including the interaction with DNA and inhibiting reproduction and cell division, disrupting the bacterial cell wall by interacting with bacterial enzymes and proteins, which are important for cell respiration and nutrients transportation. Silver with a positive charge (cationic silver [Ag^+^]) has antimicrobial activity and all silver-based dressings achieve antimicrobial effects by releasing Ag^+^ [17]–[20]. Navarro *et al.* [21] studied the interaction of silver complexes with *L. mexicana* and found that these complexes are effective in killing of the promastigote form of the parasite. A further mechanism by which silver may enhance wound healing in CL is its anti-inflammatory effects. Silver may reduce the activity of matrix metalloproteinase (MMP), which has been linked with delayed wound healing and inflammation [13]–[17].

The goal of this randomized, assessor-blind controlled clinical trial was assessment of the efficacy of either silver or non-silver containing dressings in the treatment of cutaneous leishmaniasis due to *L. major*.

## Methods

The protocol of this trial and supporting CONSORT checklist and CONSORT flow diagram are available as supporting information; see Protocol S1, Checklist S1, and Flow Diagram S1.

### Ethics statement

The protocol and consent form of the study were reviewed and approved by the Ethics Committee of the Center for Research and Training in Skin Diseases and Leprosy, Tehran University of Medical Sciences, on December 1, 2007. The study was conducted in accordance with the ethical principles provided by the Declaration of Helsinki and by ethical codes provided by the Undersecretary of Research at the Iran Ministry of Health and guidelines of ICH Good Clinical Practice (GCP). Written informed consents were obtained from patients at patient allocation. For all minor participants (younger than 18 years old), whom were recruited into the study, a written informed consent was obtained from a parent or a legal guardian.

### Design

This study was designed as a three parallel arm with 1∶1∶1 allocation ratio randomized, assessor-blind clinical trial.

### Participants

The study was conducted at Golabchi Clinic an outpatient referral clinic for CL cases in Kashan where is endemic for CL due to *L. major*. Kashan is located about 210 km south of Tehran, capital of Iran.

Inclusion criteria were: **a**) parasitologically confirmed cases of CL based on positive smear and/or culture; **b**) otherwise healthy subjects on the basis of medical history, **c**) age of 12 to 60 years; **d**) willingness to participate in the study and signing the informed consent form (by the patient or his/her parent/guardian in cases younger than 18 years).

Exclusion criteria were: **a**) pregnant or lactating women; **b**) duration of lesion more than 3 months; **c**) number of lesions more than 5; **d**) ulcer size greater than 5 cm in largest diameter; **e**) history of receiving full course standard treatment (antimonials); **f**) history of allergy to meglumine antimoniate (MA) or silver; **g**) serious systemic illnesses (as judged by the physician); **h**) participation in any drug trials in the last 60 days; **i**) indication for systemic treatment with MA; **j**) presence of secondary bacterial infection of the lesion according to clinical appearance.

Withdrawal criteria were: **a**) occurrence of a serious adverse event, or **b**) withdrawal of the consent.

### Interventions

Eligible patients were randomly allocated into three groups and treated for 6 weeks with either:

- weekly injections of intralesional (i.l.) MA (Glucantime®; Rhodia Laboratories, Rhone-Poulenc, France) alone,
- weekly injections of i.l. MA combined with application of a non-silver dressing (Atrauman®, Hartmann, CMC Consumer Medical Care GmbH, Germany) on the lesions, or
- weekly injections of i.l. MA combined with application of a silver containing dressing (Atrauman® Ag, Hartmann, CMC Consumer Medical Care GmbH, Germany) on the lesions.

Injections of MA were done by using insulin syringes with 30-G needles, 0.1 milliliter. The drug was infiltrated intradermally in each one square centimeter of a lesion, first at the circumference and then in the center if necessary, depending on the size of each lesion until blanching occurred. While originally, it was planned that the dressings applied on the lesions weekly, for a better compliance of the patients, the dressings were applied on the lesions and the patients were asked to change it every other day. Base of both dressings were similar and were made of polyester.

### Follow up

An assessor who was blinded to the type of treatment visited the patients at weekly intervals during the treatment period and 1-month and 5 months after the last treatment session.

### Outcome measures

The primary end point of this study was the clinical cure of the lesion (complete healing defined as more than 75% reduction, clinical improvement defined as 50%–75% reduction, and no response to treatment defined as less than 50% reduction in the size of the lesion compared with baseline). These end-points were assessed at the end of the treatment period (end of week 6) and 4 weeks later. The secondary end-points included treatment related side effects and relapse. The relapse defined as a reappearance of lesions at the site or periphery of previously healed lesions or an increase in the size of lesions after initial improvement was assessed 5 months after the termination of treatment.

### Sample size

Fifty-six lesions per treatment group were needed to have 80% power to detect a significant difference in the expected cure rate of 60% in the i.l. MA alone group and the desired cure rate of 85% in the i.l. MA and either silver or non-silver dressing-treated group at week 10 with a type I error level of 0.05. Compensating for a 20% loss-to-follow up, recruiting 68 (round up to 70) lesions per treatment group looked reasonable.

### Randomization

A random sequence generated by using the online software Random Sequence Generator, which is available at URL: www.random.org [22]. It was done by an investigator with no clinical involvement in the trial. R. Talaee was responsible for enrollment of the patients.

### Randomization concealment

The method for randomization concealment was to use sequentially numbered, opaque, sealed envelopes (SNOSE). The envelopes were kept in a safe box, which was only accessible to A. Khatami who was responsible for assigning the patients to the interventions.

### Blinding

An assessor who was blinded to the type of treatment visited the patients at weekly intervals during the treatment period and 1-month and 5 months after the last treatment session. The patients received their dressings from the members of the study team other than the assessors and were instructed to take off their dressings before each visit and not to explain to assessors whether they have used dressing or not.

### Statistical methods

Data obtained and recorded were entered in SPSS 17 for Windows software (SPSS Inc, Chicago, Ill, USA). Normally distributed data were reported as mean ± standard deviation (SD). If the distribution of data did not follow the normal distribution median and 25^th^ and 75^th^ percentiles were reported. An intent-to-treat analysis was performed at 2 time points (end of the treatment period [day 42] and one month later [day 72]). The proportions of complete healing, clinical improvement, and withdrawal were compared between the two groups by calculating Absolute Risk Reductions (ARR) based on the proportion of complete healing, which was defined as more than 75% reduction in the size of the lesion compared with baseline in each group. Ninety-five percent confidence intervals (95% CI) were provided. To compare the mean for data with normal distribution one-way analysis of variance (ANOVA) and for data that do not follow normal distribution proper nonparametric statistical tests were used. To compare the proportion of males and females among different groups, chi squared test was used. Fisher exact test was used to compare the rates of relapse and drug-related adverse events. A 2-sided *P*<0.05 was considered significant.

### Documentation

In each visit the characteristics of the lesions including the number, location, size of induration, ulcer, scar (greatest diameter multiplied by diameter perpendicular to it), adverse events, and concomitant treatment (if any) were recorded on the Case Report Forms (CRFs).

## Results

### Participants flow

**Figure 1:**
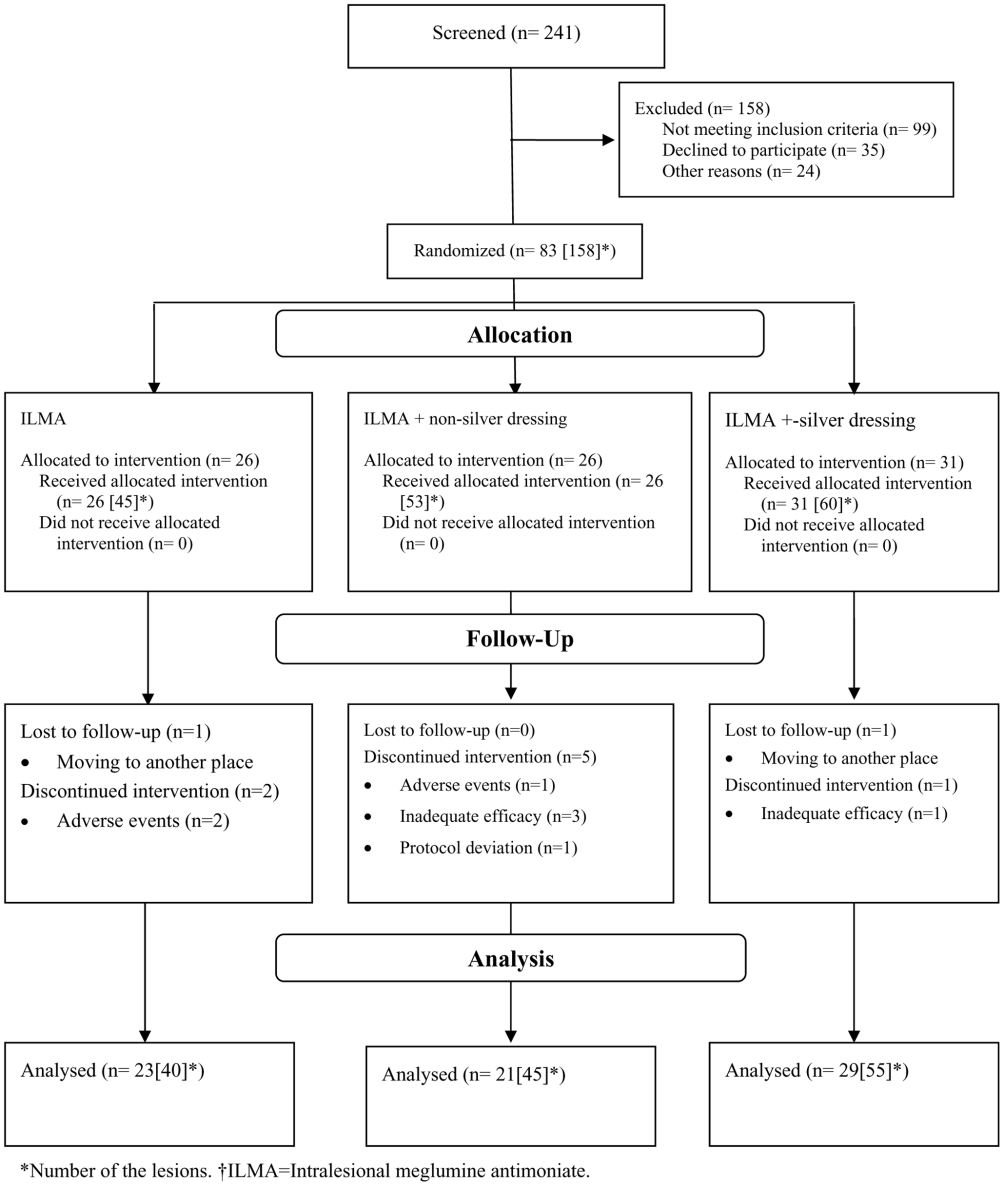
Patients' disposition flow diagram (CONSORT flowchart) from day 0 to day 72 (4 weeks after termination of treatment).

Between September 2008 and April 2010, 241 cases of cutaneous leishmaniasis were screened for the eligibility criteria of the trial and 83 cases with 158 lesions were recruited into the trial after giving written informed consents. During the follow up period, 10 patients with 18 lesions were lost to follow up or withdrawn from the study, so ten weeks after initiation of interventions, 73 patients with 140 lesions completed the trial (Figure 1).

### Baseline data

Baseline characteristics of the recruited patients and their lesions on the day of recruitment are presented in Table 1 and 2, respectively. Number of the lesions according to the number of the patients at the beginning of the study is provided in Table S1. The proportion of male patients allocated in the i.l.MA arm was significantly larger than their proportion in the i.l.MA in combination with silver dressing (Yates chi^2^ (corrected for continuity)  = 5.16, df  = 1, *P* = 0.0231).

**Table 1:** Baseline characteristics of the included patients.

Characteristic	Total (N = 83)	ILMA* (n = 26)	ILMA+ non-silver dressing (n = 26)	ILMA+ silver dressing (n = 31)
**Age (mean ± SD†) years**	28.81 (14.45)	32.88 (12.92)	30.31 (15.41)	24.13 (13.97)
**Duration of lesions (mean ± SD) weeks**	7.95 (2.67)	8.31 (2.86)	8.46 (2.15)	7.23 (2.82)
**Number of lesions (mean ± SD)**	1.90 (1.21)	1.77 (1.34)	2.00 (1.47)	1.94 (1.12)
**Male (%)**	39 (47.0)	8 (30.8)	11 (42.3)	20 (64.5)

* ILMA  =  intralesioinal meglumine antimoniate. †Standard deviation.

**Table 2:** Baseline characteristics of the included lesions.

Characteristic	Total (N = 158)	ILMA (n = 45)	ILMA+ non-silver dressing (n = 53)	ILMA+ silver dressing (n = 60)
**Induration area median (25^th^ percentile-75^th^ percentile) mm^2^**	169 (79–433)	270 (120–557)	156 (67–490)	141 (48–346)
**Ulceration area median (25^th^ percentile-75^th^ percentile) mm^2^**	6 (1–25)	8 (2–37)	6 (0–15)	6 (0–24)

* ILMA  =  intralesioinal meglumine antimoniate.

As demonstrated in Figure 1, loss to follow up resulted in the difference in total number of the lesions demonstrated in Table 2 (158 lesions), Table 3 (141 lesions), and Table 4 (140 lesions).

**Table 3:** Clinical response 6 weeks after initiation of the treatment.

	No response	Partial improvement	Complete healing	Total
**ILMA** * **(% [95% CI^†^])**	26 (65.0 [49.5–77.9])	9 (22.5 [12.3–37.5])	5 (12.5 [5.5–26.1])	40 (100)
**ILMA + non-silver dressing (% [95% CI])**	37 (80.4) [66.8–89.3]	6 (13.0 [6.1–25.7])	3 (6.5 [2.2–17.5])	46 (100)
**ILMA + silver dressing (% [95% CI])**	41 (74.6 [61.7–84.2])	7 (12.7 [6.3–24.0])	7 (12.7 [6.3–24])	55 (100)
**Total (%[95% CI])**	104 (73.8 [65.9–80.3])	22 (15.6 [10.5–22.5])	15 (10.6 [6.6–16.8])	141 (100)

* ILMA  =  intralesioinal meglumine antimoniate. † 95% Confidence Interval.

**Table 4:** Clinical response 10 weeks after initiation of the treatment.

	No response	Partial improvement	Complete healing	Total
ILMA* (% [95% CI])	17 (42.5 [28.5–57.8])	7 (17.5 [8.8–32.0])	16 (40.0 [26.4–55.4])	40 (100)
**ILMA + non-silver dressing (% [95% CI])**	25 (55.6 [41.2–69.1])	1 (2.2 [0.04–11.6])	19 (42.2 [29.0–56.7])	45 (100)
**ILMA + silver dressing (% [95% CI])**	27 (49.1 [36.4–61.9])	8 (14.5 [7.6–26.2])	20 (36.4 [24.9–49.6])	55 (100)
**Total (%[95% CI])**	69 (49.3 [41.1–57.5])	16 (11.4 [7.2–17.8])	55 (39.3 [31.6–47.6])	140 (100)

ILMA  =  intralesioinal meglumine antimoniate. † 95% Confidence Interval.

### Efficacy

Treatment results in the three different treatment groups at the end of treatment period and 4 weeks later are shown in Tables 3 and Table 4, respectively.

- *i.l.MA versus i.l.MA combined with non-silver containing polyester dressing*
- ARR (95% CI) in i.l.MA versus i.l.MA combined with the non-silver containing dressing groups was 5.98% (−7.07% to 20.25%) 6 weeks after initiation of treatment. ARR (95% CI) in i.l.MA versus i.l.MA combined with the non-silver dressing groups was −2.22% (−22.12% to 18.10%) one month later.
- *i.l.MA versus i.l.MA combined with silver containing polyester dressing*
- ARR (95% CI) between i.l.MA versus i.l.MA plus silver containing polyester dressing groups was −0.23% (−13.53% to 14.82%) 6 weeks after initiation of treatment. ARR (95% CI) between i.l.MA versus i.l.MA plus silver containing polyester dressing groups was −3.64% (−15.36% to 22.82%) 1 month later.
- *i.l.MA combined with non-silver containing polyester dressing i.l.MA combined with silver containing polyester dressing*
- ARR (95% CI) between i.l.MA combined with non-silver containing polyester dressing versus i.l.MA plus silver containing polyester dressing groups was −6.21%(−18.28% to 6.52%) after 6 weeks of treatment. ARR (95% CI) between i.l.MA combined with non-silver containing polyester dressing versus i.l.MA combined with silver containing polyester dressing groups was 5.86% (−12.86% to 24.31%) 1 month later.

### Safety

The most commonly observed adverse events were itching and burning, edema and exudation, which occurred in 17, 12 and 2 lesions, respectively. No statistically significant difference was detected in occurrence of adverse among different study arms (table 5). Five months after the end of treatment, the only case of relapse was observed in the i.l. MA plus silver dressing group.

**Table 5:** Frequency of treatment related adverse events.

	Itching and burning (%)* [%]^†^	Exudation (%) [%]	Edema (%) [%]	Dermatitis (%) [%]	Total
**ILMA** *	3 (33.3) [17.6]	1 (11.1) [50.0]	5 (55.6) [41.7]	0 (0.0) [0.0]	9 (100)
**ILMA + non-silver dressing**	8 (61.5) [47.1]	1 (7.7) [50.0]	3 (23.1) [25.0]	1 (7.7) [100]	13 (100)
**ILMA + silver dressing**	6 (60.0) [35.3]	0 (0.0) [0.0]	4 (40.0) [33.3]	0 (0.0) [0.0]	10 (100)
**Total**	17 [100]	2 [100]	12 [100]	1 [100]	32 (100) [100]

* ILMA  =  intralesioinal meglumine antimoniate. (%)  =  % in each treatment group. [%]  =  % in each adverse event group. Fisher's exact test = 0.561.

## Discussion

CL is a major health problem in many developing countries, affects hundreds of thousands of people each year, and is associated with a high disease burden at both individual and social levels in endemic areas [5], [23].

According to the best of our knowledge, there were no published studies regarding assessment of the efficacy of dressings in the treatment of CL lesions. In the consultative meeting to develop a strategy for treatment of CL at Institute Pasteur of Paris in 2006, it was recommended to use hydrating dressings (specially the types that contain polyurethane) for the treatment of the lesions of CL [13].

Although the efficacy of the i.l. MA injections alone in the treatment of lesions of CL due to *L. major*, 6 weeks after initiation of this intervention, was different from 26.0% to 86.7% in the past studies, it was higher than the observed cure rate of 12.5% in the present study [24]–[30]. The reported cure rate of 40% at week 10 after initiation of treatment in the present study is also quite low. The lower cure rate which has been found in the present study might be explained by either the differences in the inclusion and the exclusion criteria or the “primary outcome” definition.

The present study indicates that silver dressing do not make significant changes in the efficacy of the i.l. MA in the treatment of CL due to *L. major*. Navarro *et al.* [21] studied anti-leishmanial effect of silver. By comparing the affinity of DNA versus albumin to two synthetic complexes of silver, they observed a strong interaction of the complexes with DNA. The affinity of the complex one ([Ag (dpq)_ 2_] NO3) for DNA was higher than for albumin but in the case of the complex two ([Ag (dppz)_ 2_] NO3) the affinity was same for DNA and albumin. The complex one reduced growth of promastigotes of *L. mexicana* by 55% and complex two showed a leishmanicidal effect. The anti-leishmanial effect of these silver complexes was dose dependent. The authors concluded that this effect was related to interaction with parasitic DNA. Navarro *et al*. [21] used silver complexes in promastigote forms of *Leishmania* in vitro, but in the present study, silver dressing was used clinically in the patients who had the amastigote form of *Leishmania* in their macrophages. So, the differences in the kind of the parasite (*L. mexicana vs*. *L. major*), in the form of it (promastigote *vs.* amastigote), in the type of silver (silver complexes *vs.* Ag^+^) and its concentration in the wound might explain the inefficacy of silver dressing in the treatment of CL.

The rate of relapse after treatment with i.l. MA in this study was similar to the studies by Sadeghian *et al.* [26], Asilian *et al.* [27], Mujtaba and Khalid [31], and Faghihi and Tavakoli-kia [32].

### Limitations

One of the main limitations was the recruited number of the patients and consequently lesions to this study. This could be explained partly by a decrease in the number of referred CL patients to Golabchi Clinic in Kashan from certain nearby places like Natanz. Changes in the incidence of CL in the region may also contribute to this problem. Despite extending the recruitment period by almost 8 months, this limitation could not be effectively overcome. There were also some limitations in randomization and blinding in the present study. An important limitation in randomization was that the patients were randomized and all lesions on each patient were treated with the same intervention. The dependency of the lesions on the patients has not been evaluated and the findings of this study have not been adjusted for the dependency. Although adjusting for dependence might widen 95% CIs, it would be associated with no important clinical implications (i.e.: the 95% CIs already indicated no statistical significance).

To minimize information bias, blinding of the participants and the assessor at the same time is more effective than blinding of the assessor alone. Double blinding was not possible in the present study since the silver and the inert dressings were not alike exteriorly, and the interventions were different in the arms of the study. While patients were asked to remove and wash the site of the application of the dressings before coming to the clinic and not to talk about the interventions they received with the assessors, the effectiveness of blinding was not assessed systematically.

Although it seems that use of dressing can improve treatment of CL due to *L. major*, the present study could not demonstrate that use of either silver or inert dressing significantly change the efficacy of i.l. MA in the treatment, and the rate of relapse and adverse events.

## Supporting Information

Checklist S1 **Completed CONSORT 2010 checklist for the study.**
(PDF)

Flow Diagram S1 **Completed CONSORT 2010 flow diagram for the study.**
(PDF)

Protocol S1 **Protocol of the study.**
(PDF)

Table S1 Number of the lesions according to the number of the patients at the beginning of the study.
(DOCX)
