# Supplementary figures and images for: Dressings Combined with Injection of Meglumine Antimoniate in the Treatment of Cutaneous Leishmaniasis: A Randomized Controlled Clinical Trial
Source: PLoS One. 2013 Jun 24;8(6):e66123. doi: 10.1371/journal.pone.0066123 (PMC3691234; doi:10.1371/journal.pone.0066123)

## CONSORT 2010 Flow Diagram

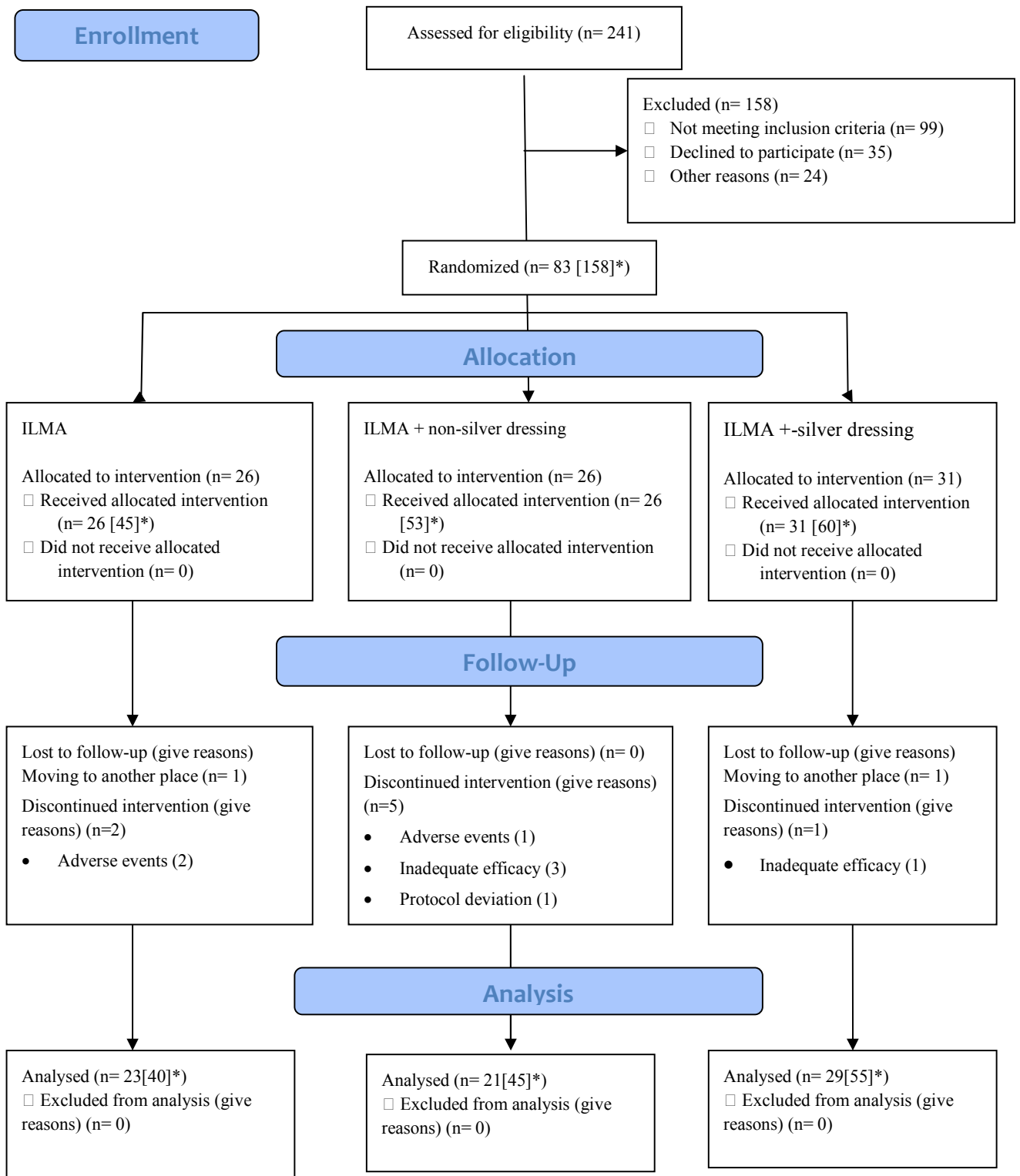

\*Number of the lesions. †ILMA=Intralesional meglumine antimoniate.

Supplement: Flow Diagram S1 — Completed CONSORT 2010 flow diagram for the study. (PDF) [file pone.0066123.s002.pdf]
