# Supplementary material for: Dressings Combined with Injection of Meglumine Antimoniate in the Treatment of Cutaneous Leishmaniasis: A Randomized Controlled Clinical Trial
Source: PLoS One. 2013 Jun 24;8(6):e66123. doi: 10.1371/journal.pone.0066123 (PMC3691234; doi:10.1371/journal.pone.0066123)
Supplement: Protocol S1 — Protocol of the study. (PDF) [file pone.0066123.s003.pdf]

Assessment of the efficacy of the combination of a silver-containing dressing with intralesional meglumine antimoniate injections in comparison with a combination of an inert dressing and intralesional meglumine antimoniate injections and intralesional meglumine antimoniate injections alone in the treatment of cutaneous leishmaniasis due to *Leishmania major*: A randomized assessor-blind controlled clinical trial

Principle Investigator

Alireza Firooz, MD-Associate Professor of Dermatology, Center for Research and Training in Skin Diseases and Leprosy, Tehran University of Medical Sciences, Tehran, Iran

Contact address: No. 415, Taleqani Avenue, Tehran 14166-13675, Iran

Tel: +98 21 88978190**, Fax:** +98 21 88963804, **Email:** firozali@sina.tums.ac.ir

Co-investigators

Alireza Khatami, MD, MSc(PH): Assistant Professor of Dermatology, Center for Research and Training in Skin Diseases and Leprosy, Tehran University of Medical Sciences, Tehran, Iran, **Email:** akhatami@tums.ac.ir

Rezvan Talaee, MD: Assistant Professor of Dermatology, Department of Dermatology, Kashan University of Medical Sciences, Kashan, Iran, **Email:** r_talaee2007@yahoo.com

Makan Rahshenas: Medical Student, Faculty of Medicine, Islamic Azad University, Tehran Medical Branch, Tehran, Iran, **Email:** makan_rah@yahoo.com

Ali Khamesipour, PhD: Associate Professor of Immunology and Microbiology, Center for Research and Training in Skin Diseases and Leprosy, Tehran University of Medical Sciences, Tehran, Iran, **Email:** khamesipour@sina.tums.ac.ir

Pedram Mehryan, MD: Dermatologist, Center for Research and Training in Skin Diseases and Leprosy, Tehran University of Medical Sciences, Tehran, Iran, **Email:** pedram_mehryan@yahoo.com

Sepideh Tehrani, MD: Assistant Professor of Dermatology, Department of Dermatology, Faculty of Medicine, Islamic Azad University, Tehran Medical Branch, Tehran, Iran, **Email:** tehrani42643@yahoo.com

Yahya Dowlati, MD, PhD: Director, Center for Research and Training in Skin Diseases and Leprosy, Tehran University of Medical Sciences, Tehran, Iran, **Email:** dowlatiy@yahoo.com

F**inancial support:** From the Vice-Chancellery of Research of Tehran University of Medical Sciences through the contract number: 132/344

Conflicts of interest: None

Expected Start Date: September 2008

Expected Termination Date (for recruitment): September 2009

List of Abbreviations

AOWCL	Acute Old World cutaneous leishmaniasis
CL	Cutaneous leishmaniasis
CR	Case Report Form
i.l.	Intralesional
MA	Meglumine antimoniate

Summary

Cutaneous leshmaniasis is a major health problem in many developing countries and affects hundreds of thousands of people each year. It is associated with a high disease burden at both individual and social levels in endemic areas. Looking for simple, inexpensive treatment methods is continued.

Unfortunately the conventional treatments of cutaneous leshmaniasis tend to concentrate only on the treatment of the microbial cause of this disease and little attention have been paid to other aspects of wound management in this disease.

This study is designed to assess of the efficacy of a combination of intralesional injections of meglumine antimoniate and non-silver dressing or silver dressing in comparison with the intralesional injections of meglumine antimoniate in the treatment of cutaneous leshmaniasis due to *L. major* through conducting a randomized ,assessor-blind controlled clinical trial.

Aim and objectives

Primary objective: To compare the proportion of cured patients between treatment group treated with intralesional injections of meglumine antimoniate MA and non-silver dressing and treatment group treated with intralesional injections of meglumine antimoniate 10 and 14 weeks of follow-up.

To compare the proportion of cured patients between treatment group treated with intralesional injections of meglumine antimoniate and silver dressing and treatment group treated with intralesional injections of meglumine antimoniate 10 and 14 weeks of follow-up.

Secondary objectives

To compare: 1) the proportion of relapsed patients 6 months after initiation of treatment, 2,) the mean time to heal of the lesions and, 3) the proportion of adverse effects during the study period in three groups.

To answer: 1) Is the combination of intralesional injections of meglumine antimoniate and non-silver dressing more effective than the intralesional injections of meglumine antimoniate in the treatment of CL due to *L. major?*, and 2) is the combination of intralesional injections of meglumine antimoniate and silver dressing more effective than the intralesional injections of meglumine antimoniate in the treatment of CL due to *L. major*?

Eligibility criteria

Eligible patients will be treated by administration of either intralesional injections of meglumine antimoniate (Glucantime®; Rhodia Laboratories, Rhone-Poulenc, France) alone, or a combination of intralesional injections of meglumine antimoniate injections with application of a dressing (Atrauman ,®Hartmann, CMC Consumer Medical Care GmbH, Germany) or a combination of intralesional injections of meglumine antimoniate injections with application of a silver containing dressing (Atrauman® Ag ,Hartmann, CMC Consumer Medical Care GmbH, Germany) on the lesions. The dressing will be applied on the lesions and will remain there for a week .Totally ,this study is expected to be conducted in 12 months. Six weeks of interventions and 6 months follow-up from the first visit of each patient is planned. The patients will be visited at weekly intervals and the characteristics of their lesions will be recorded on the Case Report Forms (CRFs) . After the 7th visit ,the patients will be visited one month later and the last visit will be on the day 180**.**

Keywords: *Leishmania majo*r, cutaneous leishmaniasis, dressing

Background

Cutaneous leishmaniasis (CL) is a major health problem in many developing countries and affects hundreds of thousands of people each year. It is associated with a high disease burden at both individual and social levels in endemic areas. Looking for simple, inexpensive treatment methods is continued .Unfortunately the conventional treatments of CL tend to concentrate only on the treatment of the microbial cause of this disease and little attention have been paid to other aspects of wound management in this disease. For example although as a rule all chronic wounds are colonized to some degrees with different microorganisms and it is well known that when the bacterial burden of the wound gradually increases, it could interfere with normal wound healing and deteriorate the inflammation and pain of the wound, in the majority of randomized clinical trials that have assessed the efficacy of different therapeutic interventions for acute OWCL, principles of wound management has been ignored. As it has been previously mentioned in literature review, using silver dressings might assist healing of the CL lesions through several mechanisms. This study is designed to assess of the efficacy of a combination of intralesional (i. l.) injections of meglumine antimoniate (MA) and silver dressing in comparison with the intralesional injections of MA in the treatment of CL due to L. major through conducting a randomized, assessor-blind controlled clinical trial

Leishmaniasis is caused by different species of intracellular protozoan ,*Leishmania* and transmitted by the bite of infected sand flies [1]. Leishmaniasis is endemic in 88 countries, mostly developing ones [4]. Ninety percent of all CL cases occurs in only 7 countries; Afghanistan, Algeria, Brazil, Iran, Peru ,Saudia Arabia and Syria [5]. Iran is endemic for CL. Almost all CL cases are caused by either *L. tropica* or *L. major*. CL has been reported from all provinces and is endemic in many of them. In some villages surrounding Kashan, cumulative

incidence of CL was estimated to be around 13.1 % in 1996 [6]. CL due to L .major usually manifests as appearance of a papule or small nodule at the site of parasite inoculation 1 to 2 months after the sand fly bite. Over a period of several weeks, the lesion will develop into an ulcer with moderate occasionally hemorrhagic exudate and crust which is accompanied by induration of the peri-ulcer skin. The skin at the periphery of the lesion is usually erythematous. In almost all cases the lesion will heal with scarring over a period of several months even in absence of treatment [1-3]. The active lesion and remaining scar can be associated with a high burden on different aspects of patient's life [11]. Many treatment modalities have been used in the treatment of CL, but, pentavalent antimonies are considered as the first line drugs for treatment of CL so far. They have to be administered only as intramuscular ,intravenous or intralesional injections and could be associated with severe side effects and significant discomfort [12]. There is no vaccine available for prevention of CL for general human use [13].A recently published systematic review demonstrated that no effective, safe and inexpensive treatment is currently available for CL [12]. It is logical to imagine that bringing all aspects of wound management paradigm into consideration for treating ulcerative lesions of CL may improve the results of any intervention that are used in the treatment of CL cases. Wound management paradigm includes treating the cause ,wound bed preparation and patient related concerns. Wound bed preparation contains debridement, control of inflammation or infection and moisture balance [14]. Unfortunately the conventional treatments of CL tend to concentrate only on the treatment of the microbial cause of this disease and little attention have been paid to other aspects of wound management in this disease. In the majority of randomized clinical trials that have assessed the efficacy of different therapeutic interventions for AOWCL, principles of wound management has been ignored [12]. Silver exerts its antimicrobial effects through several

mechanisms. The main mechanism is thought to be the interaction with DNA and inhibiting reproduction and cell division [17, 18]. Silver also can disrupt the bacterial cell wall by interacting with bacterial enzymes and proteins which are important for cell respiration and nutrients transportation [17, 19]. Silver with a positive charge (cationic silver) has antimicrobial activity [19]. All silver based dressings achieve their antimicrobial effects by releasing cationic silver (Ag+). Cationic silver binds to thiol groups and alters the normal function of proteins and leads to bacterial cell wall rupture. Bacterial cell death will occur as a result of leakage of the cell contents. Also silver ion can bind to bacterial enzymes and interfere with cell respiration and nutrient transportation [19]. Navarro et al. [22] noted that the anti-leishmanial effect of these silver complexes is related to interaction with parasitic DNA [22]. A further mechanism by which silver may enhance wound healing in CL lesions is its reported anti-inflammatory effects. Silver may reduce the activity of matrix metalloproteinase (MMP), which has been linked with delayed wound healing and inflammation [17, 21].

Goal and Objectives

Primary objective: To compare the proportion of cured patients between treatment group treated with intralesional (i.l.) injections of meglumine antimoniate (MA (and non-silver dressing and treatment group treated with intralesional (i.l (.injections of meglumine antimoniate (MA) 10 and 14 weeks of follow-up . To compare the proportion of cured patients between treatment group treated with intralesional (i.l.) injections of meglumine antimoniate (MA) and silver dressing and treatment group treated with intralesional (i.l.) injections of meglumine antimoniate (MA) 10 and 14 weeks of follow-up.

Secondary objectives

To compare:

- the proportion of relapsed patients 6 months after initiation of treatment
- the proportion of adverse effects during the study period in three groups

To answer:

- Is the combination of intralesional (i.l.) injections of meglumine antimoniate) MA) and non-silver dressing more effective than the intralesional injections of MA in the treatment of CL due to Leshmania major?
- Is the combination of intralesional (i.l.) injections of meglumine antimoniate (MA) and silver dressing more effective than the intralesional injections of MA in the treatment of CL due to *L. major*

Patients and Methods

Design and setting

This study is designed as a three parallel arm with 1:1:1 allocation ratio randomized, assessor-blind clinical trial and will be conducted in Kashan where is endemic for CL due to *L. major*. Kashan is located about 210 km south of Tehran, capital of Iran.

Inclusion criteria

a) Parasitologically confirmed cases of CL based on positive smear and/or culture

b) Otherwise healthy subjects on the basis of medical history

c) Age of 12 to 60 years

d) Willingness to participate in the study and signing the informed consent form (by the patient or his/her parent/guardian in cases younger than 18 years).

Exclusion criteria

a) Pregnant or lactating women

b) Duration of lesion more than 3 months

c) Number of lesions more than 5

d) Ulcer size greater than 5 cm in largest diameter

e) History of receiving full course standard treatment (antimonials)

f) History of allergy to meglumine antimoniate (MA) or silver

g) Serious systemic illnesses (as judged by the physician)

h) Participation in any drug trials in the last 60 days

i) Indication for systemic treatment with MA

j) Presence of secondary bacterial infection of the lesion according to clinical appearance.

Withdrawal criteria

a) Occurrence of a serious adverse event, or

b) Withdrawal of the consent

Randomization and randomization concealment

A random sequence will be generated by using the online software Random Sequence Generator, which is available at URL: www.random.org.[23] It will be done by an investigator with no clinical involvement in the trial. Dr. Talaee will be responsible for enrollment of the patients. The method for randomization concealment is to use sequentially numbered, opaque, sealed envelopes (SNOSE). The envelopes are kept in a safe box, which was only accessible to Dr. Khatami who will be responsible for assigning the patients to the interventions.

Interventions

Eligible patients will be randomly allocated into three groups and treated for 6 weeks with:

a) weekly injections of intralesional (i.l.) MA (Glucantime®; Rhodia Laboratories, Rhone-Poulenc, France) alone,
b) weekly injections of i.l. MA combined with application of a non-silver dressing (Atrauman® ,Hartmann, CMC Consumer Medical Care GmbH, Germany) on the lesions,
c) weekly injections of i.l. MA combined with application of a silver containing dressing (Atrauman® Ag, Hartmann, CMC Consumer Medical Care GmbH, Germany) on the lesions.

MA will be injected by using insulin syringes with 30-G needles, 0.1 milliliter. The drug will be infiltrated intradermally in each one square centimeter of a lesion, first at the circumference and then in the center if necessary, depending on the size of each lesion until blanching occurred. The dressings will be applied on the lesions and the patients will be asked to change it every other day. Base of both dressings are similar and are made of polyester.

Follow up

An assessor who is blinded to the type of treatment will visit the patients at weekly intervals during the treatment period and 1-month and 5 months after the last treatment session. The patients will receive their dressings from the members of the study team other than the assessors and will be instructed to take off their dressings before each visit and not to explain to assessors whether they have used dressing or not.

Documentation

In each visit the characteristics of the lesions including the number, location, size of induration, ulcer, scar (greatest diameter multiplied by diameter perpendicular to it), adverse events, and concomitant treatment (if any) will be recorded on the Case Report Forms (CRFs).

Sample size

Fifty-six lesions per treatment group were needed to have 80% power to detect a significant difference in the expected cure rate of 60% in the i.l MA alone group and the desired cure rate of 85% in the i.l. MA and either silver or non-silver dressing-treated group at week 10 with a type I error level of 0.05. Compensating for a 20% loss-to-follow up, recruiting 68 (round up to 70) lesions per treatment group will be reasonable.

Outcome measures

The primary end point of this study is the clinical cure of the lesion (complete healing is defined as more than 75% reduction , clinical improvement defined as 50%-75% reduction, and no response to treatment defined as less than 50% reduction in the size of the lesion compared with baseline). The end-points will be assessed at the end of the treatment period (end of week 6) and 4 weeks later.

The secondary end-points include treatment related side effects and relapse. The relapse is defined as a reappearance of lesions at the site or periphery of previously healed lesions or an increase in the size of lesions after initial improvement was assessed 5 months after the termination of treatment.

Statistical methods

The recorded data will be entered in SPSS 17 for Windows software (SPSS Inc, Chicago, Ill, USA). Normally distributed data will be reported as mean ± standard deviation (SD). If the distribution of data do not follow the Normal distribution, median and interquartile range will be reported. An intent-to-treat analysis will be performed at 2 time points (end of the treatment period [day 42] and one month later [day 72]). The proportions of complete healing, clinical improvement, and withdrawal will be compared between the two groups by calculating Absolute

Risk Reductions (ARR) based on the proportion of complete healing, which is defined as more than 75% reduction in the size of the lesion compared with baseline in each group. Ninety-five percent confidence intervals (95% CIs) will be provided. To compare the mean for data with normal distribution one-way analysis of variance (ANOVA) and for data that do not follow normal distribution proper nonparametric statistical tests were used. Fisher exact test was used to compare the rates of relapse and drug-related adverse events. A 2-sided $P < 0.05$ will be considered significant.

Ethical considerations

This protocol and consent form is reviewed and approved by the Ethics Committee of the Center for Research and Training in Skin Diseases and Leprosy, Tehran University of Medical Sciences, on December 1, 2007. The study will be conducted in accordance with the ethical principles provided by the Declaration of Helsinki and by ethical codes provided by the Undersecretary of Research at the Iran Ministry of Health. Informed consent (Appendix II) will be obtained from patients at patient allocation.

References

1. Vega-Lopez F, Hey RJ. Parasitic worms and protozoa. In: Burns T, Breathnach S, Cox N, Griffiths C, editors. Rook's Textbook of dermatology.7th ed. Oxford: Blackwell Science; 2004. p. 32.35-32.47.
2. Akilov OE, Khachemoune A, Hasan T. Clinical manifestations and classification of Old World cutaneous leishmaniasis. *Int J Dermal* 2007; **46**:132-42.
3. Dowlati Y. Cutaneous leishmaniasis: clinical aspect. *Clin Dermatol* 1996; 14:425-31.
4. Leishmaniasis. Tropical Disease Research, Progress 1995-96. Thirteenth Programme Report, WHO, Geneva, 1997.
5. Desjeux P. Leishmaniasis: current situation and new perspectives. *Comp Immunol Microbiol Infect Dis* 2004; **27:**305-18.
6. Nilforoushzadeh M, Sadeghian G. Cutaneous leishmaniasis (In Persian).Isfahan; Orouj Publications and Isfahan University of Medical Sciences Publications; 2001.
7. Bailey MS, Lockwood DN. Cutaneous leishmaniasis. *Clin Dermato.* 2007; **25**:203-11.
8. Reithinger R, Dujardin JC, Louzir H, Pirmez C, Alexander B, Brooker S. Cutaneous leishmaniasis. *Lancet Infect Dis* 2007; **7**:581-96.
9. Alvar J, Yactayo S, Bern C. Leishmaniasis and poverty. *Trends Parasitol* 2006; **22**:552-7.
10. Murray HW, Berman JD, Davies CR, Saravia NG. Advances in leishmaniasis. *Lancet* 2005; **366**:1561-77.
11. Yanik M, Gurel SM, Simsekt Z, Kati M. The psychological impact of cutaneous leishmaniasis. *Clin Exp Dermatol* 2004; **29**:464–7.

12. Khatami A, Firooz A, Gorouhi F, Dowlati Y. Treatment of acute Old World cutaneous leishmaniasis: a systematic review of the randomized controlled trials. *J Am Acad Dermatol*. 2007; **57**:335.e1-29.

13. Khamesipour A, Rafati S, Davoudi N, Maboudi F, Modabber F. Leishmaniasis vaccine candidates for development: a global overview. *Indian J Med Res* 2006; **123**:423-38.

14. Sibbald RG, Orsted HL, Coutts GS, Keast DH. Best practice recommendation for preparing the wound bed: update 2006. *Wound Care Canada* 2006; **4**:R6-R18.

15. Dow G. Infection in chronic wounds. In: Krasner DL, Rodeheaver GT, Sibbald RG, editors. Chronic wound care: a clinical source book for healthcare professionals. 3rd ed. Wayne, PA. Health Management Publications, Inc. 2001; p.343-56.

16. White RJ, Cooper R, Kingsley Andrew. Wound colonization and infection the role of topical antimicrobials. *Br J Nursing* 2001; **10**:578-83.

17. Graham C. The role of silver in wound healing. *Br J Nurs* 2005; **14**:S22, S24, S26.

18. Yang DJ, Quan LT, Hsu S. Topical antibacterial Agents. In: Wolverton SE, editor. Comprehensive dermatologic drug therapy. 2nd Ed. Philadelphia: Saunders Elsevier: 2007; p. 525-46.

19. Ovington LG. The truth about silver. *Ostomy Wound Manage* 2004; **50** (9A suppl): 1S-10S.

20. Vermeulen H, van Hattem JM, Storm-Versloot MN, Ubbink DT. Topical silver for treating infected wounds. *Cochrane Database Syst Rev* 2007; 1:CD005486.

21. Demling R, DeSanti L. The role of silver in wound healing. Part 1: Effects of Silver on wound management. *Wounds Suppl* 2001; **13**: 5-15.

22. Navarro J, Fajardo EJC, Marchan C. New silver polypyridyl complxes: synthesis, characterization and biologic activity on *Leishmania mexicana*. *Arzneim.-Forsch/Drug Res* 2006; 56: 600-4.

23. Random Sequence Generator. Available at: www.random.org.
