# Supplementary material for: Dressings Combined with Injection of Meglumine Antimoniate in the Treatment of Cutaneous Leishmaniasis: A Randomized Controlled Clinical Trial
Source: PLoS One. 2013 Jun 24;8(6):e66123. doi: 10.1371/journal.pone.0066123 (PMC3691234; doi:10.1371/journal.pone.0066123)
Supplement: Table S1 — Number of the lesions according to the number of the patients at the beginning of the study. (DOCX) [file pone.0066123.s004.docx]

Table S1. Number of the lesions according to the number of the patients at the beginning of the study.

| **Number of lesions** | **Number of the patients (%)** | **Number of Patients * Number of the lesions (%)** |
| --- | --- | --- |
| 1 | 42 (50.7) | 42 (26.6) |
| 2 | 24 (28.9) | 48 (30.4) |
| 3 | 6 (7.2) | 18 (11.4) |
| 4 | 5 (6.0) | 20 (12.7) |
| 5 | 6 (7.2) | 30 (19.0) |
| Total | 83 (100.0) | 158 (100.0) |
